# Assessing the role of *Porphyromonas gingivalis* in periodontitis to determine a causative relationship with Alzheimer’s disease

**DOI:** 10.1080/20002297.2018.1563405

**Published:** 2019-01-29

**Authors:** Sim K. Singhrao, Ingar Olsen

**Affiliations:** a Dementia and Neurodegenerative Diseases Research Group, Faculty of Clinical and Biomedical Sciences, School of Dentistry, University of Central Lancashire, Preston, UK; b Department of Oral Biology, Faculty of Dentistry, University of Oslo, Oslo, Norway

**Keywords:** Alzheimer’s disease, chronic periodontitis, cause, infection, *P. gingivalis*, lipopolysaccharide risk factor, intervention

## Abstract

Chronic periodontitis of 10 years’ duration is reported to become a twofold risk factor for the development of Alzheimer’s disease (AD). Periodontitis is modifiable, and this fits with the current action plan for preventing AD. However, until periodontitis, becomes acknowledged as a firm risk factor for AD, this risk will continue. Here, we put forward our own argument based on the current literature for *in vivo* infection-mediated periodontal disease models supporting the antimicrobial protection hypothesis of AD and interventional studies supporting the causal links. Oral infections with *Porphyromonas gingivalis*, or introduction of its lipopolysaccharide (LPS), in various mouse models has demonstrated the development of key neuropathological hallmark lesions defining AD. These are extracellular amyloid-beta plaques, phosphorylated tau, neurofibrillary tangles, widespread acute and chronic inflammation, blood–brain barrier defects together with the clinical phenotype showing impaired learning and spatial memory. Live *P. gingivalis* and its LPS (commercial or from ‘microbullets’) are powerful peripheral and intracerebral inflammatory signalling initiators, and this has direct implications on memory and lesion development. Maintaining a healthy oral microbiome and managing periodontal disease with regular surveillance and good oral hygiene throughout life is likely to reduce the unnecessary burden of AD in some individuals.

## Introduction

Periodontitis is a highly prevalent oral disease in humans affecting nearly 50% of the population worldwide [1]. The polymicrobial infectious aetiology of chronic periodontitis is due to the host’s sub-gingival pathobiome, which initiates hard/soft tissue destruction that worsens with advancing age. Furthermore, the dysbiotic oral biofilm consortia can affect the functioning of the brain, potentially causing depressive illnesses, as implicated in the development of dementia [2]. Risk factors for periodontal disease are smoking [3], alcohol consumption [4], and poor oral hygiene [5]. Paganini-Hill *et al*. [6] highlighted that behavioural factors involving oral hygiene were significant in dementia onset, stating that, dentate individuals who did not brush their teeth daily, had a 22–65% greater risk of developing dementia compared with individuals who brushed their teeth three times daily [6]. These statistics suggest a proportion of individuals are particularly susceptible to *Porphyromonas gingivalis* infections, because not everyone suffers from periodontitis and not all who develop Alzheimer’s dementia suffer from periodontitis [7]. Periodontal disease is modifiable by both professional intervention and personal behavioural changes associated with oral hygiene [8,9], and this offers an avenue for reducing unnecessary mental health suffering for some individuals in their old age. In addition, therapeutic elements of prevention and treatment of dementia with a view to anti-*P. gingivalis* therapy are being sought. For example, Cortexyme Inc®, a USA based company is seeking their lead compound, COR388; to treat dementia in Phase 1 clinical trials. Such preventive measures are vital for when standard periodontal therapy becomes a challenge for both the patient (the ‘vulnerable category’ of patients according to the mental health act), and the treating dentist [www.cortexyme.com. https://www.cdc.gov/chronicdisease/resources/publications/aag/alzheimers.htm]

Alzheimer’s disease (AD), the most common form of dementia, is the leading cause of cognitive and behavioural impairment worldwide [10]. As the elderly population, keeps increasing so does the incidence of AD manifesting in two different forms: familial and sporadic. The latter form is most frequent, constituting about 95% of the cases but its cause remains open to debate. Both forms have identical neuropathological hallmarks, which are accumulations of hyper-phosphorylated tau composed of neurofibrillary tangles, and extracellular amyloid-beta (Aβ) deposits called ‘amyloid plaques’. Tau protein is prone to hyper-phosphorylation at serine and threonine residues due to the activity of multiple kinase enzymes orchestrating various signalling pathways for normal and key infection-related cellular functions [11–13]. Managing AD is a financial and medical challenge worldwide and prevention via modifiable factors is one of the key ways to avoid and/or decelerate progression of this disease [8,9]. Since the acceptance of any hypothesis explaining the cause of AD must involve the two hallmark proteins (Aβ and phosphorylated-tau tangles), and interventional studies in humans having tested beneficial outcomes, we asked the question, how does sub-gingival dysbiosis under the influence of *P*. *gingivalis*, the keystone pathogen of periodontal disease, relate to the cause of AD?

The main risk factors for the sporadic form of AD, which correlates with chronic periodontitis, are advancing age and loss of up to nine teeth [14]. Another study in which patients with chronic periodontitis and/or gingivitis, were monitored for 10 years demonstrated an increased risk for dementia (1.13%) compared with the control (0.92%) group [15]. While periodontitis correlates with other inflammatory pathologies such as cardiovascular diseases [16–18] and diabetes [19], there is overlap of AD with ischemic stroke disease [20] and insulin resistance or as some scientists like to call it, type 3 diabetes [21]. Periodontal disease is modifiable, and this provides the rationale for assessing causative associations with AD. The focus here is on summarising key epidemiological studies that have established the timeline and size of risk, as well as research on *in vivo* infection with *P. gingivalis* and its LPS supporting AD clinicopathological causal links and interventional trials showing clinical benefits.

## 10-year exposure to chronic periodontitis doubles the risk for AD

An epidemiological study by Kondo *et al*. [22] provided the initial concept, which proposed that persons with premature tooth loss were a significant risk factor for AD. Gatz *et al*. [23], investigated the risk of developing AD due to tooth loss in identical twins. For this univariate human model, tooth loss around 35 years’ age provided an odds ratio of 1.74 (95% CI 1.35, 2.24) for developing AD. Stein *et al*. [14] linked periodontitis to cognitive deficit thereby proposing periodontal disease as a risk factor for developing AD later in life. They concluded that missing up to nine teeth carried the highest risk for developing late-onset AD with an odds ratio of 2.2 (95% CI 1.1, 4.5). A retrospective study conducted by Chen *et al*. [24] found a strong link between chronic periodontal disease (exposures of around 10-years) and AD and this correlates with a prospective laboratory-based study in which circulating antibodies to two oral bacteria (*Fusobacterium nucleatum* and *Prevotella intermedia*) were linked to a cognitive deficit 10 years later [25]. In summary, Tzeng *et al*. [15], Chen *et al*. [24] and Sparks Stein *et al*. [25] suggested that gingivitis, and chronic periodontal disease of 10 years duration can promote AD. Furthermore, Stein *et al*. [14] showed tooth loss due to periodontal disease can double the risk for AD onset.

## Concept of systemic inflammation contributing to memory loss

Dunn *et al*. [26] noted (from medical records), the occurrence of repeated systemic infections in elderly subjects prior to their clinical diagnosis of dementia. This led to the assumption that inflammation plays a negative role on the health of the brain. With focus on peripheral inflammation, Holmes *et al*. [27] suggested that circulating systemic inflammatory markers (cytokines) were negatively influencing memory in sporadic AD cases. Further studies examined the links with specific periodontal pathogens to the functional cognitive loss seen in clinical AD cases. To this end, Noble *et al*. [28] found that *P. gingivalis* infection was associated with impaired spatial/episodic memory in AD with an odds ratio of 2.00 (95% CI 1.19 to 3.36) after adjusting for confounders. Subsequent studies focussed on detecting acute phase inflammatory mediators in the plasma of blood taken from confirmed AD cases in relation to periodontal pathogens/periodontitis and confirmed systemic inflammatory marker contribution from oral bacteria [29–31].

Ide *et al*. [31] set out to test the hypothesis that circulating inflammatory cytokines due to periodontal disease bacteria were associated with greater rates of cognitive decline in clinical AD cases. The study recruited 59 participants with mild to moderate AD in which cognition and circulating inflammatory markers were tested. The majority of participants (52) were followed-up at 6 months when they all underwent repeat assessment of their initial biomarkers. The study revealed that the presence of periodontitis at baseline was associated with a six-fold increase in the rate of cognitive decline in participants over the 6-month follow-up period. Periodontitis at baseline was also associated with a relative increase in the pro-inflammatory state over the six-month follow-up. The authors concluded that periodontitis is associated with an increase in cognitive decline in AD. In this study, the scientific hypothesis linking cognitive decline with the body’s inflammatory responses was supported. The weaknesses of the study were the small number of participants and absence of control participants with intact cognition.

## Host's microbiome dysbiosis is an important environmental factor

A symbiotic oral microbiome plays a role in healthy living and successful ageing [32,33]. However, a pathobiome prevails where pathogenic bacteria have changed their original commensal status, as with *P. gingivalis*-associated periodontal disease being a typical example. A pathobiome in any part of a host’s body represents an environmental risk factor. Although aberrant infections such as those linking Lyme disease (*Borrelia burgdorferi* transmitted by a bite from infected ticks, and syphilis (also known as an atrophic form of general paresis caused by *Treponema pallidum*), eventually lead to AD [34,35], they represent a separate risk factor to the so-called environmental risk that is restricted to pathobiomes and lifestyle choices. This distinction is important in view of the apolipoprotein gene allele 4 (*APOE ε4*) as a susceptibility gene that interacts with environmental risk factors such as a sub-gingival pathobiome that results in periodontal disease and/or combined with smoking, poor choices of diet and a sedentary lifestyle. This will likely enhance its biological function in favour of AD. Hence, the current discussion, where possible, is limited to *P. gingivalis* infections because its interactome shows overlaps with AD susceptibility genes making this bacterium an excellent candidate for confirming the environmental risk factor status [2]. With the demonstration of *P. gingivalis* LPS exclusively in AD brains, Poole *et al*. [36] have provided the rationale for *in vivo* proof of concept studies.

## The (simplified) amyloid cascade

The insoluble Aβ deposits (amyloid plaques) in the AD brain [37] are the consequence of amyloid precursor protein (APP) proteolysis along the N terminus (start of the protein) to the cytoplasmic tail at the C terminus (end of the amino acid chain terminated by a free carboxyl group). The enzymes generating Aβ are known as beta-secretase 1 or BACE 1, which couples with γ-secretase in the familial form of AD [38–40]. BACE 1 in this context therefore, recognizes the cleavage site of the mutated (mt)APP gene in the familial form of AD and results in enhanced Aβ production [41]. This genetic trait is the basis for generating transgenic mouse models for evaluating human AD. However, APP in the sporadic form of AD is not mutated [42], and the results of infections can vary according to the genetic make-up of the host animal. This fact has to be considered by researchers when selecting animal models to test their hypothesis and by readers when comparing experimental outcomes. Whilst, Aβ40 is the most prominent species (80–90 %) found in AD brains, the amyloidogenic Aβ42, overall represents the lesser component (5–10 %) [37,43]. Other species of Aβ fibrils (Aβ39, 38, 34, 33) also occur in the AD brain but their presence is generally neglected [44] for reasons poorly understood.

## AD-transgenic mice support experimental periodontitis as a nominal risk

Currently, there is only one report that employed the APP-transgenic model (APP-Tg) carrying the Swedish and Indiana mutations [45] infected with *P. gingivalis* to assess the role of periodontitis in the development of AD hallmark pathology (Table 1). After the lead author’s communication with the senior author of reference [45], the experimental regime was clarified as the one in their published article was somewhat misleading. It is therefore time to correctly state that Ishida *et al*. [45], induced experimental periodontitis via an oral, mono-infection with live *P. gingivalis* ATCC 33277^T^. This is a seronegative strain commonly used by global scientists in laboratory investigations. The mice in this study were infected every other day with 1 × 10^9^ CFU over the first 10 days (five infections/10 days), and sacrificed five weeks after the first infection. They noted enhanced deposition of Aβ40, and Aβ42 amyloid plaques in the hippocampus and higher levels of IL-1β and TNF-α in the infected APP-Tg group compared to the control/sham-infected group. Despite showing statistically significant differences in the Aβ40 and Aβ42 amyloid plaques (determined by image analysis) and protein (determined by ELISA assay), Ishida *et al*. [45] concluded that five mono-*P. gingivalis* infections over the first 10 days of the experiment were likely to have exacerbated the disease process rather than having contributed to the overall Aβ hallmark pathology. Behavioural testing demonstrated that the cognitive function was significantly impaired in periodontitis-induced APP-Tg mice compared to the sham-infected group. A mechanistic explanation for the greater cognitive deficit in the infected APP-Tg group was an increased intracerebral inflammation following experimental periodontitis.

**Table 1.** Summary of pathological hallmark protein appearance and functional testing following introduction of *P. gingivalis* and/or LPS in experimental mice.

*In vivo* model, no. of infections and duration	Type of infection	Pathology outcome	Behavioural testing. AD-like phenotype outcome	All supporting references
Apolipoprotein E knock out (ApoE^−^^/−^) mice. 10^9^ CFU, with four infections over 12 weeks' [ref 65] and eight infections over 24 weeks' [refs 64, 66], duration	Periodontitis-induced oral, (periodontal) mono-infection with *P. gingivalis* FDC 381 ATCC 53977 [ref 63].	Inflammation (complement activation and oxidative stress) Defective hippocampal BBB	Not done	63–66
Amyloid precursor protein-transgenic (APP-Tg) mice carrying the Swedish and Indiana mutations. 10^9^ CFU, five infections over first 10 days of experiment. Entire duration of experiment was 5 weeks	Periodontitis-induced oral, mono-infection with *P. gingivalis* ATCC 33277^T^	Greater deposition of Aβ40 and Aβ42 amyloid plaques in hippocampus and levels of IL-1β and TNF-α in infected APP-Tg mice compared to control (sham-infected) APP-Tg group	Cognitive function was significantly impaired in periodontitis-induced APP-Tg mice	45
C57BL/6N wild-type (2 months old), middle-aged mice (12 months old) and age-matched cathepsin B sufficient/knockout (CatB^−^^/−^) mice inoculated with PgLPS (1 mg/kg) daily, for 5 weeks	Systemic exposure to purified *P. gingivalis* LPS (PgLPS) from ATCC 33277^T^	Neuroinflammation (activated astrocytes and microglia); intracellular Aβ in middle-aged WT mice only	Induced learning and memory deficits in middle-aged WT mice only	55
Female C57BL/6J wild-type mice C57BL/6J, at 4 and 52 weeks’ age groups 10^9^ CFU, with repeat infection every 48 h over 6 weeks	Periodontitis-induced oral, mono-infection with *P. gingivalis* ATCC 33277^T^	Inflammation as evidenced by inflammatory mediator (cytokine) release	Impaired learning and memory at middle aged (52 weeks’ group)	62
C57BL/6 wild type mice. 6 weeks' age Inoculum size was 10^9^ CFU, 3 infections/week (66 infections in all) over 22 weeks' duration	Periodontitis-induced oral, mono-infection with *P. gingivalis* (strain W83)	Inflammation, extracellular Aβ42 amyloid plaques and ser396 residue of tau protein phosphorylation and neurofibrillary tangle formation in hippocampus	N/A	72
C57BL/6, 8 week old male mice	*P. gingivalis*-LPS and various inhibitors of the TLR signalling pathway intarperitoneal single injection(s) 5 mg/kg	Induced glial cell activation and induced inflammation via synthesis of inflammatory cytokines	Learning and memory impairment initiated via TLR4 signalling pathway	74

*In vitro* findings of Mueller-Steiner *et al*. [44] with Aβ42 peptide incubation with cathepsin B (a lysosomal enzyme that can mimic the enzymatic activity of BACE 1 in wild type APP), suggests that Aβ42 can be degraded to Aβ40 and Aβ38. Taken together, the *P. gingivalis*-infected APP-Tg group of the Ishida *et al*. [45] study resulting in greater total Aβ load detected with their 82e1 antibody may have been the product of Aβ42 truncation. Therefore, Aβ oligomer distinction would have been a better way to evaluate plaque data in the infected group. Another weakness of this model was that the method of entry of *P. gingivalis* into the mouse brain remained unclarified. However, greater amounts of LPS in the infected mouse brains were recorded implying that this *P. gingivalis* virulence factor had reached the brain and was responsible for stimulating intracerebral antigen presenting cells (glia) to upregulate cytokine expression and liberation. However, functional tests provide useful causal links with the AD phenotype even in the familial form of AD.

## Mouse models with wild type APP support *P. gingivalis* as a risk factor for AD

**Figure 1.**
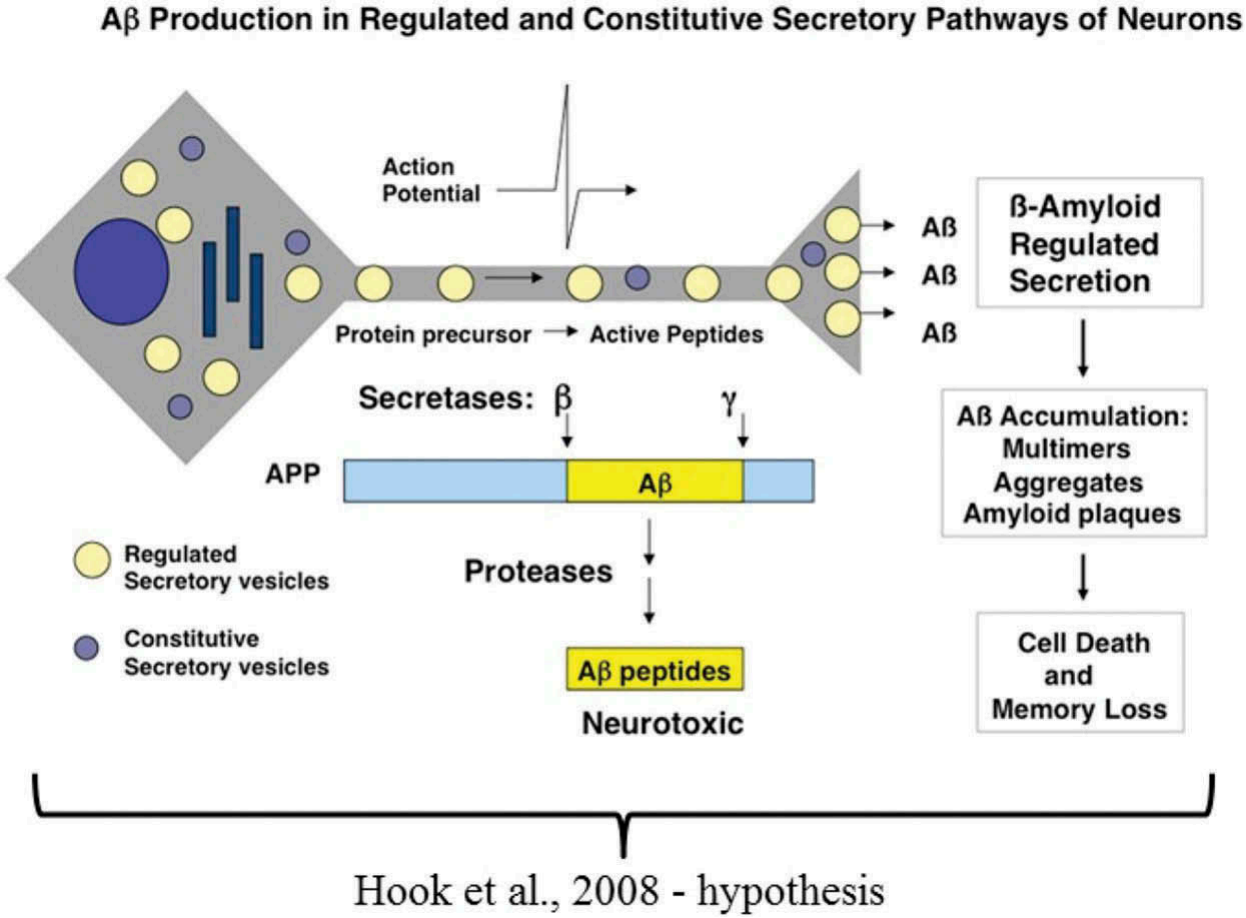
The schematic suggests that the insoluble Aβ plaques are the result of intracellular processing of the wild type amyloid precursor protein via the regulated secretory vesicle pathway mediated by cathepsin B (Taken from ref. 46 with permission).

In contrast to the familial form of AD, the sporadic form results mainly from the enzymatic processing of the wild type (wt)APP. Cathepsin B coupled with the common γ-secretase activity releases Aβ [42,46,47]. Both BACE 1 (mtAPP) and cathepsin B (wtAPP) cleave at their specific amino acid motifs [46,48–51]. The insoluble Aβ amyloid plaques are unequivocally extracellular, but it is not clear if their precursor protein processing is extracellular or intracellular or both. Hook *et al*. [46] hypothesized intracellular processing of wtAPP via the regulated secretory vesicle pathway mediated by cathepsin B and its exit from the neuron (Figure 1). Unlike the mtAPP, that implies extracellular processing by BACE 1 resulting in enhanced Aβ, cathepsin B processing of wtAPP appears to be intracellular yielding less Aβ. Neuropathology diagnosis relies on a specific threshold of Aβ amyloid plaques in both forms of AD; this suggests that the sporadic form is a result of defective clearance whilst the familial form results from enhanced deposition of this insoluble protein accounting for time differences in their early (familial) and late (sporadic) onsets. In this respect, the Takayama *et al*. [52] study links *P. gingivalis* to sleep disturbances and subversion of microglial cell function, and paves the way for Aβ build-up.

*P. gingivalis* is an intracellular bacterium that enters lysosomal bodies of cells following an endocytic entry and the trans-Golgi network via the endoplasmic reticulum to avoid immune surveillance and extend its viability [53]. Rather surprisingly, *P. gingivalis* and its LPS can manipulate endosomal/lysosomal enzymes [54] to degrade wtAPP, intracellularly (Figure 3), whilst IL-1β cytokine [55] provides the inflammophilic sustenance (Figure 3). Since IL-1β is part of the inflammasome assembly, *P. gingivalis* can modify related activity in several ways [56]. Among them are ATP/P2X7-signalling molecules, which are associated not only with periodontitis but also with the development of several systemic diseases [55,56]. Both TLR-2 and NLRP3 can recognize a functional bacterial amyloid known as curli, within Aβ amyloid plaques [57] and AD brain Aβ deposits transduce the activation of the NLRP3 inflammasome in microglial cells *in vitro* and *in vivo* [58–61].

**Figure 2.**
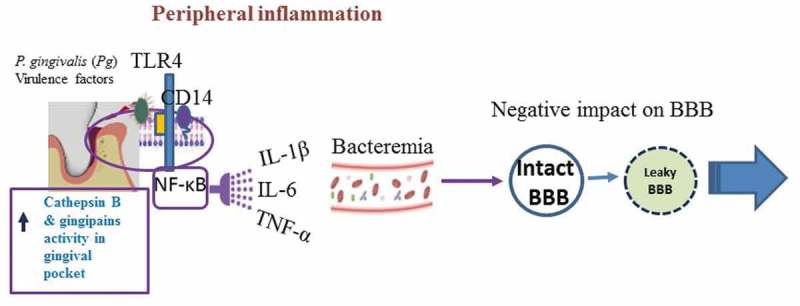
The schematic representation suggests the role of peripheral inflammation that in this example is contributed by a periodontitis pocket infected by *P. gingivalis*. NF-κB signalling gives rise to cytokines and IL-1β and TNF-α appear to weaken the blood-brain barrier (BBB) [65,66]. Bacteraemia allows *P. gingivalis* and its virulence factors to access the systemic circulation and enter the brain. *P. gingivalis* and its virulence factors enter cells and pass along the endosome/lysosomes where they encounter cathepsin B [46,55].

**Figure 3.**
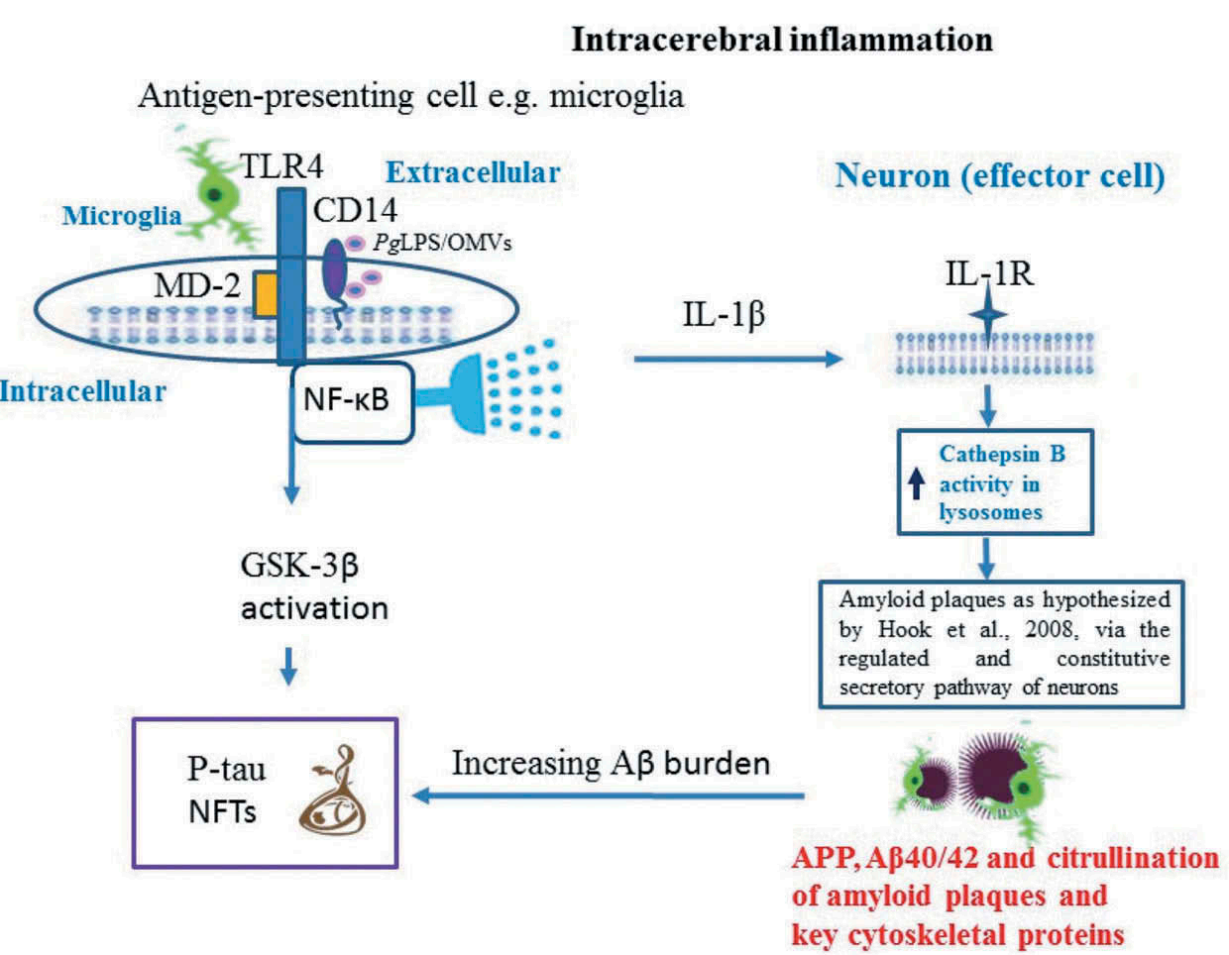
Illustrates the direct pathways by which *P. gingivalis* activity gives rise to the Alzheimer phenotype. Zhang *et al. *[74] suggest this involves toll like receptor 4 (TLR-4) signalling) and Wu *et al. *[55] suggest microglial cytokine IL-1β for neuronal function that bears its receptor on the membrane for intracellular processing of the amyloid precursor protein (APP) and amyloid beta release as hypothesized by Hook *et al.* [46]. Ilievski *et al*. [72] demonstrated the build-up, in the mice brains, of amyloid plaques outside the neurons and phosphorylation of tau on ser396 residue, an activity likely to be the result of the glycogen synthase-3β (GSK-3β) enzyme. GSK-3β can be activated via the NF-κB signalling cascade or the increasing amyloid plaque burden leading to neurofibrillary tangles (NFTs) forming. Inflammation following *P. gingivalis* entry into brain from an oral niche is supported by Poole *et al.* [64] and subsequently by Ilievski *et al.* [72]. The encapsulated *P. gingivalis* W83 strain can citrullinate structural proteins in glia and in neurons as described in reference [73].

### Wild type mouse model of experimental periodontitis supports neuroinflammation and AD phenotype according to advancing age

Ding *et al*. [62] investigated periodontal disease effects on the brain in C57BL/6J wild-type mice in four (young) and 52-week (middle-aged) groups (Table 1). Induction of experimental periodontitis was by an oral dose of live *P. gingivalis* (ATCC 33277^T^) mono-infection using 1 × 10^9^ CFU, which was repeated every 48 h over six weeks in each group. Evidence of intracerebral inflammation was obtained by inflammatory cytokine gene expression (using molecular biology methodologies), and protein release (using ELISA assay). Cytokine levels (IL-1β, IL-6 and TNF-α) by both methods were significantly higher in the *P. gingivalis* mono-infected older age group. Ding *et al*. [62] also performed behavioural tests and demonstrated statistically significant outcomes for impaired spatial learning and memory in the older age (middle-aged, 52 week) group infected with *P. gingivalis* compared to the younger (4-week old) and middle-aged uninfected mice. Increased intracerebral inflammation accounted for the impaired spatial learning and memory following experimental periodontitis. The lack of demonstration of *P. gingivalis* or its LPS entry into the mouse brain alongside omission of observations towards Aβ protein in the neuronal cell body is a weakness of the study. This may imply that any sequestered Aβ in the regulated secretory vesicle pathway generated by cathepsin B processing was below detection limits, provided the bacterium and or its LPS entered the brain. However, functional tests provide useful causal links with AD phenotype according to advancing age, but imply that this is an inflammation-mediated event secondary to infection.

### Apolipoprotein E knockout mouse model of acute and chronic periodontitis for AD neuropathology

The apolipoprotein E knockout (ApoE^−^^/−^) mouse harbours wtAPP in which periodontitis is induced following an oral *P. gingivalis* mono-infection [63] (Table 1). The mice received repeat infections (6 in 12 weeks’ duration and 12 in 24 weeks’ duration) with 1 × 10^9^ CFU. The two time points corresponded to acute and chronic periodontitis in mice. This model was the first to evaluate migration, to the brain, of the same *P. gingivalis* strain used to infect the host orally at the 24-weeks’ infection timeline, when the oral disease (periodontitis) had become chronic [63,64]. Acute phase inflammation in the form of the activated complement system was detected at 24 weeks’ post infection with evidence of neuronal vulnerability to necrotic cell death [64]. At 12 weeks’ post infection, oxidative stress and damage to the microvasculature in the hippocampus were noted [65], but subsequent blood–brain barrier (BBB) permeability in the cerebral (hippocampus) cortex became apparent only after 24 weeks [66]. These findings support the chronic nature of periodontitis that subsequently converts to risk for AD as highlighted by former prospective and retrospective studies [24,25]. Poole *et al*. [64] and Rokad *et al*. [65] endorsed the innate immune responses in the form of complement activation and reactive oxygen species (ROS)-mediated damage respectively, as described for human AD [67]. Furthermore, BBB defects [66] linked aging and AD protective barrier breakdowns [68–71]. The weaknesses of the ApoE^−^^/−^*P. gingivalis*-mono-infection model are the inability to monitor insoluble Aβ due to the total apolipoprotein gene knockout because apolipoprotein E is one of the three essential proteins required for amyloid fibril formation. However, the positive outcomes of this study, in relation to the similarity between the sporadic form of AD pathology and the ApoE^−^^/^^−^ mouse model are many. Firstly to proof of concept testing that *P. gingivalis* following bacteraemia can translocate from its oral niche to the brain [64]. Secondly, the peripheral inflammation had a negative impact on the BBB integrity [66]. Thirdly, *P. gingivalis* entry related directly to the innate immune responses impacting on intracerebral inflammation in the form of ROS, and complement activation [64,65]. These outcomes are significant findings that outweigh the inability to assess Aβ pathology in this periodontitis-AD model [63].

### Wild type mouse model of periodontitis demonstrates the cardinal AD lesions

The infection periodontal model of Ilievski *et al*. [72] is C57BL/6 wild-type mice of 6 weeks’ age (Table 1). This is the youngest cohort of animals tested in any of the infection models thus far. Induction of experimental periodontitis was by an oral dose of live *P. gingivalis* (W83), serotype 1 encapsulated, bacterial mono-infection using 1 × 10^9^ CFU, repeated every other day (Monday-Friday) of every week for 22 weeks. Evidence of intracerebral entry of the same *P. gingivalis* (W83) used to infect the host orally at the 22-weeks’ infection timeline demonstrated its intracellular presence using fluorescent in-situ hybridisation or FISH as used by Singhrao *et al*. [66]. FISH in the Ilievski *et al*. [72] mice brain sections clearly demonstrates the sites of infection in the various brain cells and in some cases the nucleus. This clearly suggests the 22-week timeline of their infection regime was somewhat excessive. However, as a proof of concept study to determine hallmark lesion formation, this is acceptable. Ilievski *et al*. [72] detected inflammation by glial cell activation supported by an elevated cytokine milieu within the hippocampus. However, most interestingly, they demonstrated the AD defining hallmark lesions. These are amyloid plaques and *de novo* phosphorylated ser396 residue in tau protein that was bound to neurofibrillary tangles within the hippocampus of the infected mice group only. Although, this study lacked functional testing in the presence of hallmark lesions in the hippocampus, it unequivocally demonstrated the causative relationship of *P. gingivalis* with emerging AD pathology, which in itself is a significant milestone.

## *P. gingivalis* can citrullinate proteins

As the Ilievski *et al*. [72] study on infections favoured *P. gingivalis* W83, which is a serotype 1 and capsulated strain, it is important to mention that such a strain has the capacity to produce citrullinated epitopes, which could be detrimental to the health of the host [73]. Currently, there are no reports of *P. gingivalis* derived peptidyl arginine deiminase (PPAD) activity in the brain possibly because these studies are still novel and because antibodies to PPAD are not widely available. In the future *P. gingivalis* W83 infections must examine this aspect of *P. gingivalis* virulence to see if the disease follows an autoimmune course or not.

## *P. gingivalis* LPS and its effect on the brain

LPS is the major surface membrane component of virulent Gram-negative bacteria such as *P. gingivalis* (Figure 3). *P. gingivalis* LPS is an important contributor to inflammation and neurodegeneration in AD because pattern recognition receptors (PRRs), like Toll-like receptors (TLRs), expressed by glia (as antigen presenting cells) can recognize pathogen-associated molecular patterns (PAMPs) in microorganisms to trigger anti-bacterial responses [45,55,62,64,74]. *P. gingivalis* LPS can stimulate CD14, TLR-2 or −4 and send signals to the nucleus by the MyD88 pathway, which initiates a cascade of events that involve an increased expression of proinflammatory cytokines [45,55,62,72,74]. This is the background to the identification of the mechanism of memory loss by the Zhang *et al*. [74] investigation discussed below. Another noteworthy feature of *P. gingivalis* is that its LPS exists in at least two different forms, O-LPS and A-LPS. The latter shows heterogeneity occurring as two isoforms, LPS_1435/1449_ and LPS_1690_ [75]. These isoforms can produce opposing effects on TLR-2 and −4 activation. The capacity to change its LPS to LPS_1435/1449_ or LPS_1690_ helps *P. gingivalis* in adjusting to the local inflammatory milieu, enabling it to survive in primary and distant sites, especially in lysosomal compartments of different tissue cell types [54].

## *P. gingivalis-*LPS model links with intracellular Aβ in cathepsin B sufficient mice

Wu *et al*. [55] (Table 1) examined the effect of systemic exposure to purified *P. gingivalis*-LPS (PgLPS) in wild-type (C57BL/6N), young (2 months old) and middle-aged (12 months old) mice and age-matched cathepsin B sufficient and knockout mice for Aβ. The experimental procedure involved intraperitoneal injection of PgLPS from ATCC 33277^T^ (1 mg/kg) daily dose, for 5 weeks. The timespan for introduction of the endotoxin was determined from their previous evaluation of systemic inflammation to induce deficits in the hippocampal long-term potentiation in middle-aged rats through microglia-mediated neuroinflammation [76]. Inflammation (activated astrocytes and microglia) and intracellular Aβ in middle-aged cathepsin B sufficient mice were reported together with learning and memory deficits. An explanation for the neuronal Aβ and the memory deficit included *P. gingivalis* using the endocytic/lysosomal pathway to enter cells and the consequence of glial cell activation and cytokine release. *P. gingivalis* LPS can mediate inflammation via cytokine (IL-1β) release, and then generate intracellular Aβ by activation of cathepsin B in an age-dependent manner [55]. This study reported AD like behaviour following *P. gingivalis* ATCC 33277^T^ LPS introduction in mice, and above all demonstrated intracellular release of Aβ according to the hypothesis of Hook *et al*. [46] via the regulated secretory vesicle pathway mediated by cathepsin B (Figure 3), which consolidated the causative relationships of *P. gingivalis* with AD [55]. This supports the Ilievski *et al*. [72] study linking extracellular Aβ amyloid plaques following *P. gingivalis* infection in mouse models expressing wtAPP. In addition, the Hook *et al*. [46] hypothesis suggested the slower accumulation of extracellular amyloid plaques in wtAPP protein. This distinction is very clear from the Ilievski *et al*. [72] images, which show fewer plaques compared with the images from the infected APP-Tg group [45].

## *P. gingivalis* LPS administration once in wild type mice supports TLR-4 signalling leading to AD phenotype

A study by Zhang *et al*. [74] aimed at dissecting out the mechanism that leads to loss of memory following *P. gingivalis* LPS intraperitoneal administration. The LPS used here is the same as that administered in other studies [55] confirming experimental consistency with previous phenotype related data outcome. The Zhang *et al*. [74] study used 8-week-old C57BL/6 mice. A single intraperitoneal injection of 5 mg/Kg *P. gingivalis*-LPS was administered with/without the TLR-4 inhibitor TAK-242. Seven days following the LPS and/or TLR-4-inhibitor challenge, cognitive tests were performed, which included the Open Field, Morris Water Maze (MWM) and Passive Avoidance, Following sacrifice, the brain tissues were examined for inflammatory markers by molecular biology, protein biochemistry and immunohistochemistry. Compared with the control group, Zhang *et al*. [74] found the test group receiving *P. gingivalis* LPS had impaired spatial learning and memory during the MWM test and a weak desire for facing fear in their ‘Passive Avoidance Test’. They also observed glial (microglia and astrocytes) cell activation in the cortex and the hippocampus regions of these mice brains alongside of upregulated cytokines (TNF-α, IL-1β, IL-6 and IL-8). The TLR-4 inhibitor (TAK-242) group mice brains demonstrated suppressed TLR-4/NF-κB signalling pathways, and the same group of animals did not show cognitive impairments. This confirmed the role of TLR-4 signalling in poor memory development.

## LPS links with tau protein phosphorylation in AD transgenic mice

Bacterial products appear to play a detrimental role in the onset and development of tau pathology. Currently, there is only one report linking tau protein phosphorylation due to *P. gingivalis* infection in mice. In another study, the AD transgenic mouse model (3xTg-AD and rTg4510) harbouring mutated tau genes [11,13] has been administered with an intraperitoneal injection of purified commercial LPS from *Escherichia coli* (12 doses over 6 weeks) in 4-months-old 3xTg-AD mice; the authors firmly implicated the role of microglial cytokines (IL-1β) in tau phosphorylation [11]. These researchers also found increased tau phosphorylation at Ser202/Thr205 and Thr231/Ser235 residues in hippocampal neurons compared with sham-infected transgenic control mice in kinase specific activity [11]. What is intriguing is that Wu *et al*. [55] have demonstrated a role for IL-1β in their cathepsin B sufficient mice following introduction of *P. gingivalis* LPS but without reporting tau phosphorylation. This leaves gaps in our knowledge as to the differing mechanisms of *E. coli* LPS and *P. gingivalis* LPS [75] in initiating innate signalling cascades.

Lee *et al*. [13] injected commercial, purified LPS from *Salmonella abortus-equi* (an aberrant infection equivalent), demonstrating that phosphorylated tau increases on select serine residues (Ser199/202 and Ser396) in the mutated (rTg4510) group of mice compared with controls. Differential tau phosphorylation at specific and plausible serine/threonine sites is acceptable as it reflects the activity of specific kinases that phosphorylate their respective amino acid residues in the tau protein [11]. This suggests that vulnerability in the tau gene, together with infections by Gram-negative bacteria, are able to influence neurofibrillary tangle formation. However, AD does not harbour mutations in the tau gene per se, and to this end, Kitazawa *et al*. [11] observed that LPS affected the phosphorylation of tau in the wild type littermates over sham-infected non-transgenic mice, which they attributed to inflammation. The results confirm a causative role of bacterial LPS in the development of the pathology in both the familial and sporadic forms of AD with inflammation playing a pivotal role in both APP processing and tau protein phosphorylation at select residues.

## Expression of AD phenotype in infected mice

Cognition describes a person’s mental ability to process information, reasoning, and learning of new skills, remembering them, and relating to them. Episodic memory, accounts for neuronal plasticity, whereby neurons can modify the patterns of connectivity of functional neurons through enhanced development of their dendrites and axon elongation [77]. This ability appears to be lost in AD and is the basis of the cognitive testing regime designed to assess new patients for dementia. Synaptic plasticity refers to the ability of synapses to modify to adapt to challenges posed by ageing and disease processes. These changes include size, morphology, density and even complete loss of synapses within defined parameters of disease [78]. Spatial memory loss correlates with synaptic loss [79] and earlier reports assigned Aβ oligomer toxicity to synaptic loss in AD [80]. *P. gingivalis* infection models described here are also displaying impaired spatial and learning and memory deficits in the younger and older age groups [55,62,74], and in the AD transgenic and wild type infected mouse models [45] where inflammation is a common feature. Therefore, inflammatory mediators such as IL-1β, which also has detrimental effect on synapses, is a likely challenge from infections that may correlate with plasticity and synapse loss resulting in cognitive dysfunctional displays by infected mice [45,55,62,74].

## Interventional studies support periodontitis as a risk factor for AD

The earliest interventional study was performed by Rolim *et al*. [81], which included 29 participants with clinically mild AD. A dentist performed a complete evaluation involving: clinical questionnaire; research diagnostic criteria for temporomandibular disorders; McGill pain questionnaire; oral health impact profile; decayed, missing and filled teeth index and complete periodontal examination before and after the intervention. The study found a reduction of orofacial pain, and improvement in the mandibular function and periodontal indices in patients with AD. These improvements were maintained until the last evaluation after 6 months and were followed by a reduction in the functional impairment due to cognitive compromise [81]. The limitation of this study is that it lacked a bigger cohort and appropriate (non-AD) controls.

## Concluding remarks

*P. gingivalis* infections and its LPS appear to be closely associated with the development of the sporadic form of AD. Data presented here are showing a consistent causative role of *P. gingivalis* infections and its LPS in mice for the development of cardinal hallmark lesions and cognitive impairment via systemic and intracerebral inflammation. In addition, inflammation is playing a pivotal role in APP processing to generate Aβ, neurofibrillary tangle formation and deteriorating memory. The time of onset (early vs late) can be explained by modes of Aβ deposition. For example, Aβ deposition in the familial AD brain may arise directly by extracellular release from mtAPP processing by BACE 1. However, wtAPP appears to undergo intracellular processing and release of Aβ mediated by the regulated secretory vesicle pathway inflicted by cathepsin B processing resulting in lesser yields. Neuropathology diagnosis relies on a specific threshold of Aβ plaques in both forms of AD; this may imply that the sporadic form has a defective clearance while the familial form has increased deposition of this insoluble protein. This suggests that the 10-year lag phase of chronic periodontitis to become a risk factor for the sporadic form of AD is plausible. Why the long lag phase? This may be due to initial weakening of the protective BBB through aging allowing easier access of bacteria into the brain. Alternatively, unlike the oral cavity, which embraces a range of diverse bacterial phylotypes and develops chronic infection only after a few weeks, the healthy brain may be slow to respond to nominally virulent (seronegative) *P. gingivalis* stains in younger human hosts because of their immune system. In contrast, seropositive 1 strains such as W83 appear to reach the brain with speed but the important feature is the bacterial load. This goes back to poor oral hygiene habits seen in patients.

Tau phosphorylation causal links with *P. gingivalis* infection completes the intrigue that *P. gingivalis* can initiate and produce both of the defining lesions of AD. Ideally, functional tests on all related periodontitis infection for AD would be desirable but not always possible, as the studies described here have shown. In all instances, live *P. gingivalis* and its LPS are powerful peripheral and intracerebral inflammatory signalling initiators, and this has direct and early implications on memory. The data presented here are significant, contributing to our growing knowledge of the causal associations between the sub-gingival pathobiome under the influence of *P. gingivalis* and development of AD. Maintaining an oral microbiome symbiosis and preventing periodontal disease with regular surveillance and good oral hygiene throughout life is likely to reduce the incidence of unwanted suffering from AD.
